# Adverse selection in a community-based health insurance scheme in rural Africa: Implications for introducing targeted subsidies

**DOI:** 10.1186/1472-6963-12-181

**Published:** 2012-06-28

**Authors:** Divya Parmar, Aurélia Souares, Manuela de Allegri, Germain Savadogo, Rainer Sauerborn

**Affiliations:** 1 Institute of Public Health, INF 324, University of Heidelberg, Heidelberg, 69120, Germany; 2 Nouna Health Research Centre, Nouna, BP 02, Burkina Faso; 3 LSE Health, London School of Economics and Political Science, Houghton Street, London, WC2A 2AE, UK

**Keywords:** Community-based health insurance, Adverse selection, Subsidy, Burkina Faso, Fixed effects

## Abstract

**Background:**

Although most community-based health insurance (CBHI) schemes are voluntary, problem of adverse selection is hardly studied. Evidence on the impact of targeted subsidies on adverse selection is completely missing. This paper investigates adverse selection in a CBHI scheme in Burkina Faso. First, we studied the change in adverse selection over a period of 4 years. Second, we studied the effect of targeted subsidies on adverse selection.

**Methods:**

The study area, covering 41 villages and 1 town, was divided into 33 clusters and CBHI was randomly offered to these clusters during 2004–06. In 2007, premium subsidies were offered to the poor households. The data was collected by a household panel survey 2004–2007 from randomly selected households in these 33 clusters (n = 6795). We applied fixed effect models.

**Results:**

We found weak evidence of adverse selection before the implementation of subsidies. Adverse selection significantly increased the next year and targeted subsidies largely explained this increase.

**Conclusions:**

Adverse selection is an important concern for any voluntary health insurance scheme. Targeted subsidies are often used as a tool to pursue the vision of universal coverage. At the same time targeted subsidies are also associated with increased adverse selection as found in this study. Therefore, it’s essential that targeted subsidies for poor (or other high-risk groups) must be accompanied with a sound plan to bridge the financial gap due to adverse selection so that these schemes can continue to serve these populations.

## Background

Over the last two decades community-based health insurance (CBHI), also referred to as micro health insurance or voluntary health insurance, has rapidly grown as a health financing tool in low and middle income countries, in areas where government or employer-based health insurance is absent. CBHI works by pooling risks and resources at the community level. The aim of such schemes is to facilitate access to healthcare and increase financial protection against the cost of illness [1-5]

Membership in most CBHI schemes is voluntary. Atim [6], Criel [7], Carrin [8], Preket et al. [9] have pointed out that voluntary membership can make these schemes vulnerable to adverse selection. Adverse selection results when high-risk or sick individuals are more likely to buy health insurance than the low-risk or healthy individuals. In the presence of adverse selection, the premiums which are fixed at the average risk in the population are not enough to cover all the claims. Hence, the financial sustainability of the scheme is jeopardized [10,11]. To limit adverse selection some schemes have restricted enrollment to the group level. Group can be defined as a household, firm, school etc. Group enrolment ensures that all individuals in the group enroll which includes both high- and low-risk individuals, reducing the risk of adverse selection.

Adverse selection has been studied extensively in the context of high-income countries [12-16]. Most of the research is focused on employer or government insurance schemes. There are few studies from low and middle income countries that have analyzed adverse selection in CBHI schemes in detail. The evidence so far is mixed. Wang et al. [17] found the presence of adverse selection in the Rural Mutual Health Care in China. Noterman et al. [18] studied a prepayment scheme for hospital care in Zaire and found adverse selection among women in their reproductive age. On the other hand, Dror et al. [19] examined the Micro Health Insurance Units in Philippines and concluded that there was no adverse selection as the morbidities among the insured and uninsured was same as concluded by De Allegri et al. [20] for the CBHI scheme in Burkina Faso. Resende and Zeidan [21] also did not find adverse selection in the Brazilian individual health insurance market.

Most of these studies are based on cross-sectional data and therefore have not studied whether the process of adverse selection changed over time. One exception is the study by Zang and Wang [22] that analyzed the New Corporative Medical scheme in China and found that adverse selection persisted in the subsequent enrolments but did not become worse over time and even displayed a trend of decline, although this trend was not significant. Another exception is the study by Wang et al. [17] of the Rural Mutual Health Care in China. They found that even though enrollment in the scheme was restricted to households this was not strictly enforced and 1/3^rd^ of the households were partially enrolled. Adverse selection mainly occurred in these partially enrolled households.

Premium subsidy is a mechanism that can mitigate adverse selection. This is because premium subsidy by reducing the cost of buying health insurance attracts individuals with low risks [23]. However, in the case of targeted subsidy, the impact on adverse selection is not clear. After subsidy if high-risk individuals from the targeted group enroll more than others, adverse selection will increase. However, if high-risk individuals are already enrolled from this group and the subsidy encourages the low-risk individuals to enroll, adverse selection will reduce. Even though targeted premium subsidies are increasing talked about in the context of CBHI schemes, we did not find any study that has captured the effect of these subsidies on adverse selection.

Hence, despite the popularity of CBHI schemes in low and middle income countries, evidence on adverse selection is limited and very few have studied the change in adverse section over time. Moreover, evidence on the effect of premium subsidies on adverse selection is completely missing. This study sets to fill these gaps in knowledge. We first examined the change in adverse selection over time and second, we evaluated the effect of targeted subsidies on adverse selection.

## Methods

### Research setting and the CBHI scheme

Burkina Faso is one of the poorest countries in the world with 43% of its population living below the poverty line. According to The World Bank [24], Burkina Faso has the lowest adult and youth literacy rates in sub-Saharan Africa. About 90% of its labor force is engaged in subsistence agriculture that contributes about 30% to the national GDP. Modern infrastructure is rare and concentrated mainly in the capital Ouagadougou. Less than 5% of its 92,000 km of roads are paved [25]. Our study took place in the Nouna Health District (NHD) located in northwest Burkina Faso, about 300 km from Ouagadougou. It is a dry orchard savannah region with 230,000 inhabitants of whom 10% live in Nouna town, the district capital.

A CBHI scheme, “*Assurance Maladie à Base Communautaire*”, was introduced in a portion of NHD where a Demographic Surveillance System (DSS) has been operating since 1993. CBHI was introduced in three phases between 2004 and 2006, following a clustered-randomized control trial (CRCT) implementation design. The study area, spread over 41 villages and Nouna town, was divided into 33 clusters: 24 rural (villages) and 9 urban (town of Nouna). Small neighboring villages that shared common ethnic and kin ties were grouped together to form a single cluster. Each year, 11 randomly selected clusters were progressively offered the opportunity to enroll into CBHI. Since 2006, all 33 clusters have been offered the opportunity to enroll in CBHI every year. The trial is described in more details elsewhere [26].

Enrolment in CBHI is voluntary. To limit adverse selection the unit of enrolment is set as a household. In addition, three months waiting period is enforced during which the enrollees are not entitled to receive CBHI benefits. Although the unit of enrolment is the household, the annual premium is set on an individual basis: 1500 CFA (2.29€) for an adult and 500 CFA (0.76€) for a child (less than 15 years old). The premium was set based on feasibility and willingness-to-pay (WTP) studies previously conducted in this region [27,28]. The premium for the entire household is paid in one single installment, at the beginning of the year, after the harvest. Membership is renewed yearly. The benefit package includes a wide range of first- and second-line medical services available within the NHD. The enrolled are asked to seek care at a pre-assigned first-line facility and only if referred they can access services at the District Hospital in Nouna. There are no copayments, deductibles or ceiling on the benefits.

Even though the enrolment increased gradually, it has remained low over time, especially among the poor households. De Allegri et al. [29] and Dong et al. [30] highlighted that the poor households found it difficult to pay for the scheme. Dong et al. [30] also found financial barriers as one of the main reasons why households did not renew their membership. Based on these findings, in 2007, it was decided to offer a 50% premium subsidy to the poor households. The poor households were identified by means of a community wealth-ranking (CWR) exercise described in detail by Souares et al. [31]. In brief, CWR entailed four steps in every village. First, a Focus Group Discussion (FGD) was conducted with the villagers to identify elements defining wealth and poverty to create wealth categories accordingly. The participants together with the village administrators and traditional leaders also identified three key informants (KI) who knew the households well. Second, each KI individually ranked all the households according to the wealth categories defined by the FGD. Third, the KIs shared their ranking with each other. In case of conflicting ranking for one household, the KIs discussed their decision until they came to a consensus. Households ranked as the bottom 20% were regarded as poor. Since 2007, CWR has been conducted every two years. The poor households identified continue to be offered subsidized premiums: 750 CFA (1.14€) for an adult and 250 CFA (0.38€) for a child.

### Data

This study used data from four rounds (2004–2007) of the Nouna Health District Household Survey (NHDHS). The survey is described in detail by De Allegri et al. [26]. In brief, 990 households i.e. 30 households per cluster were randomly included in the NHDHS, approximately 7900 individuals or 10% of the population. The DSS provided the sampling frame. The analysis presented here included only those individuals who were offered CBHI in a particular year.

Every year, the NHDHS field team interviews the household members of these 990 households and collects data on demographic and socio-economic indicators, self-reported morbidity, health care seeking behavior, insurance membership, and perceptions about the quality of health services.

### Analytical model

To study adverse selection, we wanted to estimate the influence of health status on insurance status, after controlling for all other variables. A fixed effects (FE) linear probability model, that took advantage of the panel nature of the sample i.e. repeated observations, was used. A linear probability model was preferred as it can be used to estimate fixed effects without losing a lot of sample, as would be the case with a fixed effects logit model.

Our panel model can be described as:

(1) $$Insurance_{it+1}=f\;\left(Z_{i}.\beta_{1i}+X_{\mathrm{it}}.\beta_{2i}+HS_{\mathrm{it}}.\beta_{3}+Y_{t}.\beta_{4}\right)\;$$

where *i = 1,…,n* represents individuals and *t = 4,5,6,7* represents years. *Insurance*_*it+1*_ is a binary choice variable that denotes the insurance status of the individual; *HS*_*it*_ denotes the health status of the individual at the time of purchasing health insurance. *Z*_*i*_ is a vector of time-invariant individual characteristics (like religion and sex) and *X*_*it*_ is a vector of time-varying observed characteristics (like age and household size); *Y*_*t*_ is a set of year dummies that capture time shocks.

Observed *Z*_*i*_ was not explicitly included because in a FE model, individual-specific dummy variables were created that captured all time-constant variation. The effect of health status on insurance status was captured by *β*_*3*_*,* after controlling for *X*_*it*_ time-varying variables and all observed and unobserved time-constant variables.

To study the change in adverse selection over time, we included an interaction term for health insurance and year. To study the effect of subsidy on adverse selection, we re-ran the FE regression with an interaction term for subsidy and health status. Hence, the FE regression was done twice with different interaction terms.

Even though the unit of enrolment was restricted to a household to limit adverse selection, this rule was not strictly followed. There were instances when some members in the household enrolled while others did not. Therefore, in line with earlier studies [17,22] we used individuals as our unit of analysis.

### Variables

The definitions of the variables and their summary statistics are shown in Table 1.

**Table 1:** Definition and descriptive statistics of independent variables

**Variables**	**Definition**	**Percentage (%) or Mean**				
		**2004**	**2005**	**2006**	**2007**	**Average**
n ^a^	No. of individuals	2878	4360	5725	5517	-
Health Status						
Sick	1 if sick ^b^; 0 otherwise*	17.9	19.2	19.4	19.3	19.1
Age (years)						
≤ 15	Age 15 years or less	45.4	42.5	40.5	38.4	41.2
16-59	Age between 16–59 years*	47.6	50.8	52.2	53.7	51.6
60+	Age 60 years or older	6.9	6.7	7.4	7.9	7.3
Education						
Literate	1 if can read/write; 0 otherwise*	31.7	37.2	40.8	43.8	39.4
Subsidized ^c^						
Subsidy	1 if given subsidy in insurance premium; 0 otherwise*	-	-	-	18.3	5.3
Household size						
Size	Number of individuals in the household	11.9	11.0	12.1	12.4	11.9
SES						
LowSES	Household SES below 33.33^th^ percentile*	34.0	32.1	33.4	33.3	33.1
MidSES	Household SES between 33.33^th^ and 66.67^th^ percentile	33.5	32.9	33.2	33.4	33.2
HighSES	Household SES above 66.67^th^ percentile	32.5	35.0	33.3	33.3	33.6
Year						
2004	Year 2004*	-	-	-	-	15.6
2005	Year 2005	-	-	-	-	23.6
2006	Year 2006	-	-	-	-	31.0
2007	Year 2007	-	-	-	-	29.8

^a^ Insurance was offered in a phased manner. From 2006 everyone was eligible for insurance.

^b^ Individual was considered sick if (s)he had an illness for at least 3 months at the time of the survey.

^c^ Subsidy was offered only in year 2007.

* Reference category for multivariate analysis.

### Dependent variable: insurance status

We created a binary choice dependent variable that depicted the insurance status of the individual for every year (1 = individual enrolled in the scheme; 0 = individual not enrolled in the scheme).

### Independent variables

#### Health status

We created a variable, *sick*, to predict an individual’s health status. Every household member, 10 years or older, was asked if (s)he was suffering from any illness that was continuing for more than three months at the time of the survey. For individuals younger than 10 years, their caretaker (preferably their mother) was asked.

#### Subsidy

To measure the effect of the subsidized premiums a binary variable, *subsidy*, was created. (1 = individuals offered premium subsidy; 0 = individual not offered premium subsidy).

#### Year

To capture time shocks, year dummies were included.

#### Interaction terms

To measure the change in adverse selection over time, the interaction term, *sick X year* was created. To measure the effect of subsidy on adverse selection, the interaction term, *sick X subsidy* was created.

#### Other variables

In addition to health status, other individual and household level variables were also included – household socio-economic status (SES), household size, education and age of the individual. Household SES was estimated by using principal components analysis (PCA) [32]. Household ownership of durable goods (plough, bicycle, radio, television and telephone) and livestock (poultry, sheep, goat, cattle, donkey, pig and horse) were used in the PCA. Households were divided into three groups (low SES, mid SES and high SES). It was assumed that households with higher SES would have higher enrolment because they could better afford to pay for CBHI, compared to lower SES households. A continuous variable, *size,* was included to measure household size since previous research of De Allegri et al. [29] and Dong et al. [30] had identified a link between household size and both enrolment and renewal rates.

### Ethical approval

The study protocol was approved by the Ethical Committee of the Medical Faculty of the University of Heidelberg, Germany (130/2002), and by the Nouna Ethical Committee in Nouna, Burkina Faso.

## Results

Data is described in Table 2. The sample of individuals increased during 2004–06, as more clusters were offered CBHI as part of the CRCT. Enrolment remained low, between 4–6.3%, but increased gradually. There was a steep increase in enrolment in 2007 when 218 (62.5%) new individuals enrolled. Dropouts (defined as individuals not renewing their membership in the following year) were high. Dropouts were highest in 2006 when 77 out of 201 i.e. 38% of those who enrolled in 2005 did not renew their membership.

**Table 2:** Description of the data

**Year**	**Dropouts**	**Newly insured**	**Re-insured**	**Total insured**^**a**^	**Total (n)**^**b**^
2004	-	126	-	126 (4.38%)	2878
2005	23	103	98	201 (4.61%)	4360
2006	77	123	113	236 (4.12%)	5725
2007	43	218	131	349 (6.33%)	5517

^a^ These numbers correspond to the insured individuals covered by the household survey. The population enrolment rates were 4.5%, 5.0%, 3.9% and 6.1% for years 2004 to 2007.

^b^ Refers to the total number of individuals offered insurance who were interviewed in the household survey.

Table 2 gives the mean or percentage for categorical variables for all independent variables. Every year 17.9% to 19.4% of the individuals were sick. The number of individuals in the younger age group decreased as the sample become older. On an average 39.4% of the individuals were literate. The household size remained stable at about 12 individuals per household. In this sample 18% of the individuals were offered premium subsidy in 2007.

Table 3 gives the mean or percentage for categorical variables for all independent variables by insurance status. Every year, a higher percentage of sick individuals were found in the insured group compared to the uninsured group. However, this difference was not found to be significant in 2004–06 suggesting that there was weak evidence for the presence of adverse selection during this time. However, in 2007 the insured group had significantly higher percentage of sick individuals providing strong evidence for adverse selection.

**Table 3:** Descriptive statistics of independent variables by insurance status

**Variables**	**Percentage (%) or Mean**											
	**2004**			**2005**			**2006**			**2007**		
	**I = 0**	**I = 1**	**p-value**	**I = 0**	**I = 1**	**p-value**	**I = 0**	**I = 1**	**p-value**	**I = 0**	**I = 1**	**p-value**
n	2752	126	-	4159	201	-	5489	236	-	5168	349	-
Health status												
Sick	17.8	20.6	0.412	19.1	22.4	0.242	19.3	22.0	0.301	18.8	26.7	0.000
Age (years)												
≤15	45.4	45.2	0.968	42.6	41.3	0.718	40.5	40.3	0.949	38.6	36.4	0.415
16-59	47.6	47.6	0.997	50.9	50.2	0.868	50.2	50.4	0.586	53.7	53.3	0.884
60+	6.9	7.1	0.930	6.6	8.5	0.294	7.3	9.3	0.246	7.7	10.3	0.082
Education												
Literate	31.4	39.7	0.050	37.1	41.3	0.223	40.2	54.2	0.000	43.3	52.1	0.001
Subsidized												
Subsidy	-	-	-	-	-	-	-	-	-	17.3	31.8	0.000
Household size												
Size	11.8	14.0	0.005	10.9	13.0	0.000	11.9	17.5	0.000	12.4	12.0	0.408
SES												
LowSES	34.9	13.5	0.000	33.1	7.0	0.000	34.7	3.4	0.000	33.8	26.1	0.000
MidSES	33.7	30.2		32.6	40.3		33.5	27.5		33.9	25.2	
HighSES	31.4	56.3		34.3	52.7		31.8	69.1		32.3	48.7	

I = 0 denotes uninsured individuals and I = 1 denotes insured individuals.

Refer to Table 2 for variable definitions.

With regard to other variables, the age composition of the insured and uninsured groups was similar although the insured group had slightly more individuals above 60 years than the uninsured one. The insured groups had more percentage of individuals who were offered subsidy than the uninsured group (31.8% vs. 17.3%). In 2004 about 13% of individuals in the insured group were from low SES households. The following two years there were even less individuals from low SES households in the insured group (7% and 3.4%). In 2007, this percentage significantly increased to 26%. Before 2007, household size of individuals in the insured group was significantly larger than the uninsured group. Throughout this period, the insured group had a higher percentage of literate than the uninsured group.

### FE results

FE results are shown in Table 4. Column 1 shows the results when interaction term *sick X year* was included (model 1) and column 2 shows the results when interaction term *sick X subsidy* was included (model 2). Referring to the interaction terms in model 1, adverse selection was the same during period 2004–2006. In 2007 adverse selection increased i.e. probability of sick individuals enrolling into CBHI increased (coefficient = 0.021). The interaction term in model 2, *sick X subsidy*, is positive and highly significant (coefficient = 0.048), implying that sick individuals who were offered subsidy had a higher probability to enroll compared to sick individuals who were not offered subsidy.

**Table 4:** FE results

**Variables**	**(1)**		**(2)**	
	**Sick x Year**		**Sick x Subsidy**	
	**Coefficient**	**SE**	**Coefficient**	**SE**
Age (years)				
≤15	0.004	0.009	0.005	0.009
≤60+	0.015	0.036	0.018	0.036
Education				
Literate	−0.001	0.006	−0.002	0.006
Subsidized				
Subsidy	0.100	0.011***	0.090	0.012***
Household size				
Size	−0.002	0.001***	−0.002	0.001***
SES				
MidSES	0.015	0.006***	0.015	0.006***
HighSES	0.028	0.007***	0.028	0.007***
Year				
2005	0.003	0.003	0.002	0.003
2006	−0.002	0.003	−0.001	0.003
2007	0.009	0.004**	0.013	0.004***
Sick X Year^a^				
Sick	0.001	0.010	-	-
Sick x 2005	0.000	0.009	-	-
Sick x 2006	0.008	0.009	-	-
Sick x 2007	0.021	0.011**	-	-
Sick X Subsidy^b^				
Sick	-	-	0.008	0.007
Sick x Subsidy	-	-	0.048	0.027*
No. of observations	18480		18480	
No. of individuals	6713		6713	
F statistic (p-value)	11.47 (0.000)		13.22 (0.000)	
R^2^	0.0078		0.0079	

Dependent variable: Insurance status dummy variable.

^a^ Interaction term only included in Model 1.

^b^ Interaction term only included in Model 2.

Refer to Table 2 for variable definitions.

***1%, **5% and *10% significance levels.

With regard to other variables, individuals from smaller households and from households who were offered subsidy were more likely to enroll. Individuals from low SES households were less likely to enroll. Year 2007 was associated with increased enrolment. Education and age were not associated with CBHI status.

## Discussion

Our study represents one of the very few attempts that captured the change in adverse selection over time in the context of CBHI. Additionally we provide possibly the first empirical evidence on the impact of targeted subsidies on adverse selection.

Our study found weak evidence of adverse selection before 2007, but strong evidence of adverse selection the following year. This is in line with an earlier study conducted by De Allegri et al. [20] who found no evidence of adverse selection in 2004. However, there are two points of differences that should be mentioned. Unlike the earlier study, our point estimates showed that the insured group had a higher percentage of sick individuals as compared to uninsured group for all the years, although this difference was not found to be statistically significant for 2004–06. However the earlier study found an equal percentage of sick individuals in both the groups. Second, our study reported about 19% individuals as sick much less than the 65% reported by the earlier study. These differences could be because the earlier study was conducted at the household level where the whole household was considered sick if any one member in the household reported being sick while we did the analysis at the individual level.

Mandatory enrolment can completely avoid the problem of adverse selection. It has been implemented in Ghana and Rwanda however the current situation in many other low income countries makes it almost impossible to implement in the near future. When mandatory enrolment is not an option other measures can be taken. Group enrolment is one such measure to reduce the risk of adverse selection. As found by Wang et al. [17] for the Rural Mutual Health Care in China adverse selection mainly occurred in the partially enrolled households. If group enrolment is properly enforced, adverse selection can be reduced as it will ensure that all group members, sick and healthy, enroll. However, group enrolment may not entirely eliminate adverse select as high-risk groups may be more attracted to voluntary CBHI (e.g. households with many members with a chronic illness may enroll more). Other measures that can further limit adverse selection like reducing the time period for enrolment and enforcing a waiting period during which CBHI benefits are not available can be implemented. These measures reduce the likelihood of buying insurance at a time when one of the family members falls sick [7].

For the CBHI scheme in Nouna, even though enrolment was restricted to a household, this rule was not strictly enforced and we found that there were partially enrolled households. Due to lack of appropriate data it was not possible to ascertain whether adverse selection was primarily due to these households. With regard to other measures a waiting period (3 months) was applied however the enrolment period in some years extended to 3 months. By reducing the enrolment period and enforcing household enrolment adverse selection could be reduced.

Our study also demonstrated that the poor individuals who were offered subsidy had a higher probability of being sick. Since the subsidy was offered in 2007, this explains the sudden spike in adverse selection seen that year. As the poor lack access to clean water, sanitation and adequate nutrition they are more likely to face higher health risks [33]. This correction between poverty and ill health was also reflected in the community perception of poverty. In the FGDs carried out during the CWR process the criteria used to differentiate between the poor and the rich included, among others, health status and capacity to pay for medical costs [34]. These two criteria are directly related to adverse selection. Hence, the poor households identified for subsidy had more sick individuals than the other households (22.94% vs. 18.49%; p-value = 0.001) probably because continued inability to seek health care when needed ultimately translates into more acute ill health.

This finding raises important concerns for CBHI schemes. CBHI is an important mechanism for increasing access to health care and providing financial protection against the cost of illness to low-income rural and informal sector workers who are currently excluded from any government financing mechanisms. Primarily reason to introduce subsidies in CBHI is to make it affordable for the poorer sections of the society. However, as found in this study, these subsidies can also increase adverse selection. Unlike private for-profit schemes, this is not really ‘adverse’ selection but rather ‘preferred’ selection in the context of CBHI. From a public health viewpoint, we want the high risk individuals to be able to benefit from health care. Nonetheless, by increasing adverse selection, targeted subsidies also put greater strain on the financial viability of the scheme.

CBHI schemes can introduce cross-subsidization (the rich households pay a higher premium) as a means to bridge this financial gap. However, in Nouna district and many other low-income regions where such schemes are operating, rich households are not necessarily rich, rather simply less poor. If premiums are increased, enrolment among these households will decline. This is likely not only to worsen equity but also to further increase adverse selection. If targeted subsidies are implemented as a means to reach universal coverage government or donor funds that subsidize premiums for high-risk populations are essential as echoed by several others [6,8,22,35].

Technical inputs for the design, management and monitoring of voluntary CBHI schemes are essential to save these schemes from problems of adverse selection [7]. Policies on subsidies that balance the objectives of universal coverage and adverse selection should be thoroughly researched as reiterated by Carrin et al. [36] who note that public authorities and donors should study these policies in terms of volume, timing and destination. Targeted subsidies for poor (or other high-risk groups) must be accompanied with a sound plan to bridge the financial gap due to adverse selection so that these schemes can continue to serve these populations.

### Study limitations

It is worth noting some limitations of this study. This study was based on a small sample of enrolled individuals, reflective of low enrolment rates at the population level. This problem was made worse due to high attrition in the sample. The random sample originally consisted of 990 households comprising of approximately 7900 individuals. Our study was based on 6713 individuals and all these individuals were not present all years. Most of this attrition could be attributed to emigration that ranged between 7-9% during this period [37]. Small sample could have biased the regression results.

## Conclusion

Adverse selection is an important concern for any voluntary health insurance scheme. In the context of CBHI which serves primarily poor populations, this problem is even more severe. Targeted subsidies are often used as a tool to pursue the vision of universal coverage, which are also associated with increased adverse selection. This study highlights the need for well researched subsidy policies that balances the objectives of universal coverage and adverse selection.

## Competing interests

The author(s) declare that they have no competing interests.

## Authors’ contributions

DP carried out the statistical analysis, interpreted the data and drafted the manuscript. AS and MDA contributed to the design of the scheme, acquisition of data, and helped in interpretation of data. GS helped in the design of the scheme and acquisition of data. RS contributed to the conceptualization and design. AS, MDA, GS and RS also critically commented on the manuscript. All authors approved the final manuscript.

## Pre-publication history

The pre-publication history for this paper can be accessed here:

http://www.biomedcentral.com/1472-6963/12/181/prepub

## References

[B1] Ekman B Community-based health insurance in low-income countries: a systematic review of the evidence Health Policy Plan 2004 19 5 249 270 10.1093/heapol/czh031 15310661

[B2] Gnawali DP Pokhrel S Sie A Sanon M De Allegri M Souares A Dong H Sauerborn R The effect of community-based health insurance on the utilization of modern health care services: evidence from Burkina Faso Health Policy 2009 90 2–3 214 222 19036467 10.1016/j.healthpol.2008.09.015

[B3] Ranson MK Reduction of catastrophic health care expenditures by a community-based health insurance scheme in Gujarat, India: current experiences and challenges Bull World Health Organ 2002 80 8 613 621 12219151 PMC2567585

[B4] Schneider P Why should the poor insure? Theories of decision-making in the context of health insurance Health Policy Plan 2004 19 6 349 355 10.1093/heapol/czh050 15459160

[B5] Schneider P Hanson K Horizontal equity in utilisation of care and fairness of health financing: a comparison of micro-health insurance and user fees in Rwanda Health Econ 2006 15 1 19 31 10.1002/hec.1014 16145721

[B6] Atim C Contribution of mutual health organizations to financing, delivery, and access to health care 1998 Abt Associates Inc, Synthesis of research in nine West and Central African countries Bethesda MD

[B7] Criel B District-based Health insurance in sub-Saharan Africa. Part I: From theory to practice 1998 ITG Press, Antwerp

[B8] Carrin G Community based health insurance schemes in developing countries : facts, problems and perspectives. FER/EIP Discussion Paper. No.1 2003 World Health Organization, Geneva

[B9] Drechsler D Jutting JP Preker AS, Zweifel P, Schellekens OP Six regions, one story Global marketplace for private health insurance: strength in numbers 2010 The World Bank, Washington, DC 29 105

[B10] Culyer AJ The dictionary of health economics 2010 2 Edward Elgar Publishing Limited, Cheltenham, UK

[B11] Cutler DM Zeckhauser RJ Richard J Culyer AJ, Newhouse JP The anatomy of health insurance Handbook of Health Economics Volume 1 2000 1 Elsevier Inc, Amsterdam, The Netherlands 563 643

[B12] Neudeck W Podczeck K Adverse selection and regulation in health insurance markets J Health Econ 1996 15 4 387 408 10.1016/S0167-6296(96)00488-2 10164036

[B13] Pauly MV Overinsurance and Public Provision of Insurance: The Roles of Moral Hazard and Adverse Selection The Quarterly Journal of Economics 1974 88 1 44 62 10.2307/1881793

[B14] Sapelli C Vial B Self-selection and moral hazard in Chilean health insurance J Health Econ 2003 22 3 459 476 10.1016/S0167-6296(02)00121-2 12683962

[B15] Savage E Wright DJ Moral hazard and adverse selection in Australian private hospitals: 1989–1990 J Health Econ 2003 22 3 331 359 10.1016/S0167-6296(02)00104-2 12683956

[B16] Simon KI Adverse selection in health insurance markets? Evidence from state small-group health insurance reforms J Public Econ 2005 89 9–10 1865 1877

[B17] Wang H Zhang L Yip W Hsiao W Adverse selection in a voluntary rural mutual health care health insurance scheme in China Soc Sci Med 2006 63 5 1236 1245 10.1016/j.socscimed.2006.03.008 16635541

[B18] Noterman JP Criel B Kegels G Isu K A prepayment scheme for hospital care in the Masisi district in Zaire: a critical evaluation Soc Sci Med 1995 40 7 919 930 10.1016/0277-9536(94)00162-M 7792631

[B19] Dror DM Soriano ES Lorenzo ME Sarol JN Azcuna RS Koren R Field based evidence of enhanced healthcare utilization among persons insured by micro health insurance units in Philippines Health Policy 2005 73 3 263 271 10.1016/j.healthpol.2004.11.018 16039345

[B20] De Allegri M Kouyate B Becher H Gbangou A Pokhrel S Sanon M Sauerborn R Understanding enrolment in community health insurance in sub-Saharan Africa: a population-based case–control study in rural Burkina Faso Bull World Health Organ 2006 84 11 852 858 17143458 10.2471/blt.06.031336 PMC2627536

[B21] Resende M Zeidan R Adverse selection in the health insurance market: some empirical evidence Eur J Health Econ 2010 11 4 413 418 10.1007/s10198-010-0219-5 20145970

[B22] Zhang L Wang H Dynamic process of adverse selection: evidence from a subsidized community-based health insurance in rural China Soc Sci Med 2008 67 7 1173 1182 10.1016/j.socscimed.2008.06.024 18653269

[B23] Selden T Premium subsidies for health insurance: excessive coverage vs. adverse selection J Health Econ 1999 18 6 709 725 10.1016/S0167-6296(99)00031-4 10847931

[B24] Things you didn't know about Africa http://go.worldbank.org/IH6NLHRGY0

[B25] Country profile: Burkina Faso http://go.worldbank.org/U6XAAQ44R0

[B26] De Allegri M Pokhrel S Becher H Dong H Mansmann U Kouyate B Kynast-Wolf G Gbangou A Sanon M Bridges J Step-wedge cluster-randomised community-based trials: An application to the study of the impact of community health insurance Health Research Policy and Systems 2008 6 1 10 10.1186/1478-4505-6-10 18945332 PMC2583992

[B27] Dong H Mugisha F Gbangou A Kouyate B Sauerborn R The feasibility of community-based health insurance in Burkina Faso Health Policy 2004 69 1 45 53 10.1016/j.healthpol.2003.12.001 15484606

[B28] Dong H Kouyate B Cairns J Mugisha F Sauerborn R Willingness-to-pay for community-based insurance in Burkina Faso Health Econ 2003 12 10 849 862 10.1002/hec.771 14508869

[B29] De Allegri M Sanon M Sauerborn R "To enrol or not to enrol?": A qualitative investigation of demand for health insurance in rural West Africa Soc Sci Med 2006 62 6 1520 1527 10.1016/j.socscimed.2005.07.036 16169645

[B30] Dong H De Allegri M Gnawali D Souares A Sauerborn R Drop-out analysis of community-based health insurance membership at Nouna, Burkina Faso Health Policy 2009 92 2–3 174 179 19394105 10.1016/j.healthpol.2009.03.013

[B31] Souares A Savadogo G Dong H Parmar D Sié A Sauerborn R Using community wealth ranking to identify the poor for subsidies: a case study of community-based health insurance in Nouna, Burkina Faso Health & Social Care in the Community 2010 18 4 363 368 20180867 10.1111/j.1365-2524.2009.00905.x

[B32] Vyas S Kumaranayake L Constructing socio-economic status indices: how to use principal components analysis Health Policy Plan 2006 21 6 459 468 10.1093/heapol/czl029 17030551

[B33] World Health Organization Commission on Macroeconomics and Health Macroeconomics and health: investing in health for economic development 2001 World Health Organization, Geneva

[B34] Savadogo G Utilization of community wealth ranking to identify the poorest households for subsidies in enrolment to community-based health insurance in Nouna, Burkina Faso 2009 Centre de Recherche en Sante in Nouna, Nouna, Burkina Faso 10.1111/j.1365-2524.2009.00905.x 20180867

[B35] Jakab M Krishnan C Community involvement in health care financing: impact, strengths and weaknesses: a synthesis of literature HNP Discussion Paper 2001 World Bank, Washington, DC

[B36] Carrin G Waelkens MP Criel B Community-based health insurance in developing countries: a study of its contribution to the performance of health financing systems Trop Med Int Health 2005 10 8 799 811 10.1111/j.1365-3156.2005.01455.x 16045467

[B37] Sie A Louis VR Gbangou A Muller O Niamba L Stieglbauer G Ye M Kouyate B Sauerborn R Becher H The Health and Demographic Surveillance System (HDSS) in Nouna, Burkina Faso, 1993–2007 Global Health Action 2010 3 5284 10.3402/gha.v3i0.5284 PMC2940452 20847837

